# Anxiety Symptoms Assessed by the Goldberg Anxiety and Depression Scale, Lifestyle Habits, and Cardiometabolic Risk in Spanish Workers

**DOI:** 10.62641/aep.2356

**Published:** 2026-10-15

**Authors:** Lucía Garrido Sepúlveda, Pedro Juan Tárraga López, María Teófila Vicente-Herrero, José Alberto Garrido, Ángel Arturo López-González, José Ignacio Ramírez-Manent

**Affiliations:** ^1^Home Hospitalization Unit, Arnau de Vilanova Hospital, 46015 Valencia, Spain; ^2^Faculty of Medicine, University of Castilla La Mancha (UCLM), 02008 Albacete, Spain; ^3^ADEMA-University School, University of the Balearic Islands, 07009 Palma, Spain; ^4^Abascal Legal Medicine Group, 28003 Madrid, Spain; ^5^Primary Care, Balearic Islands Health Service, 07010 Palma, Spain; ^6^Faculty of Medicine, University of the Balearic Islands, 07120 Palma, Spain

**Keywords:** anxiety, Goldberg Anxiety and Depression Scale, cardiometabolic risk, occupational health, metabolic syndrome

## Abstract

**Background:**

Anxiety has been increasingly recognized as a relevant contributor to cardiometabolic dysfunction through neuroendocrine, inflammatory, and behavioral mechanisms. However, evidence evaluating the relationship between anxiety symptoms, lifestyle habits, and cardiometabolic risk in large occupational populations remains limited. This study aimed to analyze the association between Goldberg Anxiety and Depression Scale (GADS) scores, lifestyle behaviors, and cardiometabolic risk factors in a large cohort of Spanish workers.

**Methods:**

A cross-sectional observational study was conducted in 119,336 Spanish workers undergoing routine occupational health examinations between 2021 and 2023. Anxiety symptoms were assessed using the GADS. Lifestyle variables included smoking status, physical activity assessed with the International Physical Activity Questionnaire (IPAQ), and adherence to the Mediterranean diet evaluated through the Prevention with Mediterranean Diet (PREDIMED) questionnaire. Anthropometric measurements, blood pressure, lipid profile, fasting glucose, obesity, abdominal obesity, and metabolic syndrome were analyzed. Multivariable logistic regression models were used to estimate adjusted odds ratios (ORs) for cardiometabolic abnormalities according to anxiety status and lifestyle factors.

**Results:**

Elevated GADS scores were significantly associated with worse cardiometabolic profiles in both sexes, including higher blood pressure, triglycerides, fasting glucose, body mass index, waist circumference, and lower high-density lipoprotein cholesterol concentrations. Workers with elevated anxiety presented markedly higher prevalences of hypertension, hypertriglyceridemia, impaired fasting glucose, abdominal obesity, and metabolic syndrome (all *p* < 0.001). After multivariable adjustment, elevated anxiety remained independently associated with hypertension (OR 6.48; 95% confidence interval (CI) 5.89–7.09), hypertriglyceridemia (OR 5.31; 95% CI 4.60–6.02), and impaired fasting glucose (OR 5.23; 95% CI 4.64–5.83). Physical inactivity and low adherence to the Mediterranean diet were associated with a less favorable cardiometabolic profile among participants with elevated anxiety scores. Elevated GADS scores were independently associated with all major cardiometabolic outcomes after multivariable adjustment, with the strongest associations observed for hypertension, hypertriglyceridemia, and impaired fasting glucose.

**Conclusions:**

Elevated GADS scores are strongly associated with adverse cardiometabolic profiles in Spanish workers, particularly when combined with unhealthy lifestyle behaviors. These findings support the integration of mental health assessment and lifestyle interventions into occupational cardiometabolic prevention strategies.

## Introduction

Cardiometabolic diseases remain the leading cause of mortality worldwide and constitute one of the greatest challenges for modern public health systems. The progressive increase in obesity, metabolic syndrome, type 2 diabetes mellitus, arterial hypertension, and atherosclerotic cardiovascular disease has been closely linked to profound lifestyle changes associated with urbanization, sedentary behavior, psychosocial stress, and unhealthy dietary habits [1, 2]. Although traditional cardiovascular risk factors are well established, increasing attention has recently been directed toward the influence of psychological factors such as anxiety, depression, chronic stress, and psychological distress, as well as behavioral determinants including physical inactivity, smoking, and unhealthy dietary patterns, on metabolic health. These factors have been associated with obesity, insulin resistance, metabolic syndrome, type 2 diabetes, and cardiovascular disease [3, 4, 5, 6, 7].

In this context, anxiety has emerged as a clinically relevant but often underestimated factor associated with cardiometabolic dysfunction. Beyond its psychiatric implications, anxiety has been linked to autonomic dysregulation, chronic activation of the hypothalamic–pituitary–adrenal axis, systemic inflammation, endothelial dysfunction, insulin resistance, and adverse health behaviors, all of which contribute to the development and progression of cardiovascular disease [4, 5, 6]. Current evidence suggests that anxiety is associated with an increased risk of cardiovascular disease and metabolic disorders, including coronary artery disease, stroke, hypertension, and metabolic syndrome, independently of conventional cardiovascular risk factors. These associations have been supported by narrative reviews, systematic reviews with meta-analyses, and large population-based studies [5, 6, 7, 8].

The relationship between anxiety and cardiometabolic risk appears to be bidirectional and multifactorial. Current evidence indicates that anxiety is associated with an increased risk of metabolic syndrome and cardiovascular disease and may contribute to unhealthy lifestyle behaviors, including tobacco use, which further exacerbate cardiometabolic risk [6]. Conversely, obesity and metabolic disturbances themselves may contribute to the onset or worsening of anxiety symptoms through inflammatory, neuroendocrine, and psychosocial pathways [9]. This complex interaction suggests that mental health and metabolic health should not be evaluated as isolated entities but rather as interdependent dimensions within a broader biopsychosocial framework.

Among lifestyle factors, physical inactivity and poor dietary quality have consistently shown strong associations with both anxiety and cardiometabolic disease. Sedentary behavior is associated with increased cardiovascular morbidity and mortality, whereas regular physical activity exerts protective effects on endothelial function, insulin sensitivity, autonomic balance, and psychological well-being [10, 11]. Similarly, adherence to the Mediterranean diet has been associated with lower incidence of obesity, metabolic syndrome, diabetes, cardiovascular disease, and depressive and anxiety symptoms [12, 13, 14]. The Mediterranean dietary pattern, characterized by high consumption of fruits, vegetables, legumes, whole grains, olive oil, fish, and nuts, appears to exert anti-inflammatory and antioxidant effects that may simultaneously improve mental and metabolic health [15].

Recent evidence has further strengthened the concept that lifestyle behaviors may mediate or modulate the relationship between psychological distress and cardiometabolic risk. Individuals with elevated anxiety levels frequently exhibit lower adherence to healthy dietary patterns, increased emotional eating, poorer sleep quality, and lower levels of physical activity [16, 17, 18]. Emotional eating in particular has been associated with abdominal obesity, insulin resistance, and adverse metabolic profiles, especially among individuals exposed to chronic stress or anxiety [19]. Consequently, the combined evaluation of anxiety and lifestyle factors may provide a more comprehensive assessment of cardiometabolic vulnerability than isolated biomedical parameters alone.

Occupational populations represent a particularly relevant setting for investigating these interactions. Working adults are exposed to multiple psychosocial stressors, including high job demands, time pressure, irregular schedules, sedentary occupations, and work–life imbalance, all of which may adversely affect both mental and physical health [20]. Moreover, workplace health surveillance programs provide a unique opportunity to identify early cardiometabolic alterations in large populations using standardized and cost-effective approaches. Despite this, mental health variables are still insufficiently incorporated into routine occupational cardiovascular risk assessment.

The Goldberg Anxiety and Depression Scale (GADS) is a simple, validated, and widely used screening instrument designed for rapid detection of anxiety and depressive symptoms in clinical and epidemiological settings [21]. Due to its brevity and ease of administration, the GADS is particularly suitable for large occupational cohorts. Although previous studies have reported associations between anxiety symptoms and cardiovascular or metabolic outcomes, several important gaps remain. Most investigations have focused on specific cardiometabolic variables, clinical populations, or psychiatric outcomes, with limited consideration of lifestyle-related factors that may influence these associations. Furthermore, few large-scale occupational studies have simultaneously evaluated anxiety symptoms, physical activity, adherence to the Mediterranean diet, smoking habits, and a broad spectrum of cardiometabolic risk indicators within the same analytical framework. Therefore, the present study extends previous research by providing a comprehensive assessment of the relationships between anxiety symptoms, lifestyle behaviors, and multiple cardiometabolic outcomes in a large cohort of Spanish workers.

In recent years, several investigations have highlighted the growing importance of integrating behavioral and psychosocial determinants into cardiovascular prevention strategies [22]. Multicomponent lifestyle interventions combining physical activity, dietary modification, and behavioral strategies have demonstrated beneficial effects on cardiometabolic outcomes, while addressing psychosocial factors may further enhance cardiovascular prevention approaches [23]. Consequently, identifying workers with unfavorable psychological and lifestyle profiles may facilitate earlier and more individualized preventive strategies.

The present study aims to evaluate the association between anxiety measured through the Goldberg Anxiety and Depression Scale, lifestyle behaviors—including physical activity, adherence to the Mediterranean diet, and smoking—and cardiometabolic risk factors in a large cohort of Spanish workers. We hypothesized that elevated anxiety scores would be associated with an adverse cardiometabolic profile and that unhealthy lifestyle behaviors would be associated with a greater cardiometabolic burden among workers with elevated anxiety symptoms. By exploring these interactions in a large occupational setting, this study seeks to contribute to a more integrated understanding of the connections between mental health, lifestyle, and metabolic risk.

## Materials and Methods

### Study Design and Population

A cross-sectional observational study was conducted in a large cohort of Spanish workers who underwent routine occupational health assessments between January 2021 and December 2023, corresponding to the participant recruitment and data collection period. Participants were recruited from different occupational sectors, including administrative, industrial, commercial, transportation, and healthcare settings, as part of periodic workplace medical surveillance programs performed throughout Spain. The study was designed following the recommendations of the Strengthening the Reporting of Observational Studies in Epidemiology (STROBE) statement for observational research [24].

The initial database comprised more than 150,000 Spanish workers who underwent routine occupational health examinations between 2021 and 2023. Of these, 1274 declined to participate in the study, 3982 lacked the required biochemical parameters, 1584 presented implausible anthropometric values, 12,725 had missing lifestyle-related data, and 11,099 did not complete the psychological questionnaires. After applying these exclusion criteria, the final analytical sample consisted of 119,336 workers (71,787 men and 47,549 women) aged 20–69 years.

All participants provided written informed consent authorizing the anonymous use of their health information for epidemiological research purposes. The study protocol complied with the ethical standards established in the Declaration of Helsinki and received approval from the Balearic Islands Research Ethics Committee (CEI-IB 4383/20).

### Sociodemographic and Lifestyle Variables

Demographic information included age, sex, educational level, and occupational social class. Educational attainment was categorized into elementary school, high school, or university education [25]. Social class was classified according to occupational category into class I (managerial/professional), class II (intermediate occupations), and class III (manual workers and basic occupations) [26] . The collection of these data was carried out by clinical interview by the health personnel who carried out the medical examinations.

Smoking status was categorized as current smoker or non-smoker. Former smokers were included in the non-smoker category due to the absence of standardized information regarding smoking cessation duration.

Physical activity was evaluated using the short version of the International Physical Activity Questionnaire (IPAQ-SF), a validated 7-item instrument widely employed in epidemiological studies to assess physical activity performed during the previous 7 days [27]. The questionnaire estimates weekly energy expenditure expressed as metabolic equivalent task minutes per week (MET-min/week). Participants were classified according to the International Physical Activity Questionnaire (IPAQ) scoring protocol into low, moderate, or high physical activity categories.

Adherence to the Mediterranean diet was assessed using the validated 14-item Prevention with Mediterranean Diet (PREDIMED) questionnaire developed for Spanish populations [28]. The questionnaire provides a total score ranging from 0 to 14 points, with higher scores indicating greater adherence to the Mediterranean dietary pattern. Consistent with previous studies, scores ≥9 points were considered indicative of high adherence to the Mediterranean diet [29].

### Assessment of Anxiety

Anxiety symptoms were assessed using the anxiety subscale of the GADS, a validated brief screening tool widely used for the detection of anxiety symptoms in primary care and epidemiological settings [30]. The questionnaire consists of nine dichotomous (yes/no) items exploring symptoms such as nervousness, excessive worry, irritability, sleep disturbances, and somatic manifestations of anxiety experienced during recent weeks.

Each affirmative response was scored as one point, resulting in a total anxiety subscale score ranging from 0 to 9. Based on previous validation studies and commonly applied criteria, a score ≥4 was considered indicative of clinically relevant anxiety symptoms [30, 31]. The questionnaire was self-administered during occupational health examinations under standardized conditions supervised by trained healthcare personnel.

### Anthropometric and Clinical Assessment

Anthropometric measurements were obtained by trained professionals following standardized international procedures. Body weight and height were measured with participants barefoot and wearing light clothing. Body mass index (BMI) was calculated as weight divided by height squared (kg/m^2^) [32].

Waist circumference was measured midway between the lower rib margin and the iliac crest using a flexible non-elastic tape [33]. Blood pressure was measured in the seated position after at least 10 minutes of rest using calibrated automatic sphygmomanometers OMRON-M3 monitor (OMRON, Osaka, Japan). Two measurements were obtained, and the average value was used for analysis [34].

### Biochemical Determinations

Venous blood samples were collected after an overnight fast of at least 12 hours. Serum concentrations of total cholesterol, high-density lipoprotein cholesterol (HDL-c), low-density lipoprotein cholesterol (LDL-c), triglycerides, and fasting glucose were determined using standardized enzymatic methods in accredited clinical laboratories participating in external quality control programs.

Cardiometabolic alterations were defined according to contemporary international clinical practice guidelines for hypertension, dyslipidaemia, and disorders of glucose metabolism:

Hypertension: systolic blood pressure ≥140 mmHg and/or diastolic blood pressure ≥90 mmHg or antihypertensive treatment [35].

Hypercholesterolemia: total cholesterol ≥200 mg/dL [36].

Elevated LDL-c: LDL-c ≥130 mg/dL [36].

Hypertriglyceridemia: triglycerides ≥150 mg/dL [36].

Low HDL-c: <40 mg/dL in men and <50 mg/dL in women [36].

Impaired fasting glucose was defined as fasting plasma glucose levels of 100–125 mg/dL, whereas participants receiving antidiabetic treatment were considered to have altered glycemic status according to current American Diabetes Association (ADA) criteria [37].

Metabolic syndrome was defined according to the harmonized international criteria proposed by Alberti *et al*. [38] as the presence of at least three of the following five components: abdominal obesity, elevated triglyceride levels, reduced HDL-cholesterol, elevated blood pressure, and impaired fasting glucose. Abdominal obesity was defined using sex-specific waist circumference thresholds (≥102 cm in men and ≥88 cm in women).

### Statistical Analysis

Normality of continuous variables was assessed using the Kolmogorov–Smirnov test together with visual inspection of histograms.

Variables with a normal distribution were compared using Student’s *t*-test, whereas variables showing non-normal distributions were analyzed using the Mann–Whitney U test. Categorical variables were compared using chi-square tests.

Comparisons between groups according to anxiety status were performed using the appropriate statistical test according to data distribution.

Sex-stratified analyses were additionally conducted due to known sex-related differences in cardiometabolic profiles and anxiety prevalence.

Multivariable logistic regression models were constructed to estimate adjusted odds ratios (ORs) and 95% confidence intervals (CIs) for cardiometabolic alterations associated with elevated Goldberg anxiety scores and lifestyle factors. Adjustment variables included age, sex, educational level, social class, smoking status, physical activity, and adherence to the Mediterranean diet.

Additional analyses were performed to evaluate the combined associations of anxiety symptoms, sedentary behavior, smoking, and poor dietary adherence with cardiometabolic risk.

All statistical analyses were performed using SPSS version 30.0 (IBM Corp., Armonk, NY, USA). A two-tailed *p*-value < 0.05 was considered statistically significant.

## Results

### Sociodemographic and Lifestyle Characteristics

A total of 119,336 Spanish workers were included in the final analysis, comprising 71,787 men (60.2%) and 47,549 women (39.8%), with a mean age of 39.6 ± 10.3 years. Table 1 summarizes the main sociodemographic, lifestyle, anthropometric, and biochemical characteristics of the study population according to sex. Men exhibited significantly higher values for body weight, waist circumference, blood pressure, triglycerides, and fasting glucose, whereas women showed more favorable HDL-c concentrations and healthier lifestyle patterns, including greater adherence to the Mediterranean diet and higher levels of physical activity. Significant sex-related differences were also observed in smoking prevalence, educational level, and occupational social class distribution, highlighting the importance of sex-stratified analyses in the evaluation of cardiometabolic risk and anxiety-related factors within occupational populations.

**Table 1.** **Sociodemographic, lifestyle, anthropometric, and biochemical characteristics of the study population according to sex**.

Variables	Men (n = 71,787) Mean ± SD / n (%)	Women (n = 47,549) Mean ± SD / n (%)	*p*-value
Age (years)	39.8 ± 10.3	39.3 ± 10.2	<0.001
Weight (kg)	81.1 ± 13.9	65.4 ± 13.2	<0.001
Height (cm)	174.0 ± 7.1	161.2 ± 6.6	<0.001
Body mass index (kg/m^2^)	26.8 ± 4.1	25.2 ± 5.0	<0.001
Waist circumference (cm)	87.7 ± 9.2	73.9 ± 7.9	<0.001
Systolic blood pressure (mmHg)	124.4 ± 15.1	114.4 ± 14.7	<0.001
Diastolic blood pressure (mmHg)	75.4 ± 10.6	69.6 ± 10.3	<0.001
Total cholesterol (mg/dL)	195.8 ± 38.9	193.9 ± 36.6	<0.001
HDL-c (mg/dL)	51.0 ± 7.0	53.7 ± 7.6	<0.001
LDL-c (mg/dL)	120.3 ± 37.7	122.6 ± 37.2	<0.001
Triglycerides (mg/dL)	123.8 ± 88.5	88.2 ± 46.5	<0.001
Fasting glucose (mg/dL)	88.1 ± 12.9	84.1 ± 11.6	<0.001
Current smokers	26,633 (37.1)	15,596 (32.8)	<0.001
Physically inactive	38,980 (54.3)	22,867 (48.1)	<0.001
Mediterranean diet adherence	29,514 (41.1)	24,300 (51.1)	<0.001
High Goldberg Anxiety and Depression Scale score	15,578 (21.7)	6086 (12.8)	<0.001
Low Goldberg Anxiety and Depression Scale score	56,209 (78.3)	41,463 (87.2)	<0.001
Elementary education	43,934 (61.2)	24,583 (51.7)	<0.001
High school education	24,336 (33.9)	19,400 (40.8)	<0.001
University education	3517 (4.9)	3566 (7.5)	<0.001
Social class I	3805 (5.3)	3281 (6.9)	<0.001
Social class II	12,634 (17.6)	15,834 (33.3)	<0.001
Social class III	55,348 (77.1)	28,434 (59.8)	<0.001

SD, standard deviation; HDL-c, high-density lipoprotein cholesterol; LDL-c, low-density lipoprotein cholesterol. Continuous variables are expressed as mean ± standard deviation and categorical variables as percentages. Comparisons between sexes were performed using Student’s *t*-test for continuous variables, according to data distribution, and chi-square tests for categorical variables. Statistical significance was established at *p* < 0.05. Physically inactive: low physical activity according to the IPAQ-SF classification. Mediterranean diet adherence: Prevention with Mediterranean Diet (PREDIMED) score ≥9 points. High Goldberg Anxiety and Depression Scale score: Goldberg Anxiety and Depression Scale score ≥4 points.

### Goldberg Anxiety and Depression Scale and Cardiometabolic Parameters

Workers with elevated anxiety scores demonstrated significantly worse cardiometabolic profiles compared with individuals presenting lower GADS scores. These differences were observed consistently in both men and women.

Among men, high anxiety scores were associated with:

higher systolic and diastolic blood pressure, elevated total cholesterol and LDL-c, markedly increased triglyceride concentrations, higher fasting glucose levels, and lower HDL-c concentrations.

Similar findings were observed among women, although the relative increase in LDL-c and total cholesterol associated with elevated anxiety appeared even more pronounced than in men.

Hypertriglyceridemia showed the greatest relative difference between anxiety groups. Men with elevated anxiety presented triglyceride concentrations almost twice as high as those with lower scores, whereas women with high anxiety exhibited triglyceride levels approximately 40% higher.

Likewise, fasting glucose concentrations were significantly higher among anxious individuals independently of sex, suggesting a relevant relationship between anxiety symptoms and altered glucose metabolism.

Detailed biochemical comparisons according to anxiety status and sex are presented in Table 2.

**Table 2.** **Cardiometabolic parameters according to Goldberg Anxiety and Depression Scale status and sex**.

Variables	Low GADS Men (n = 56,209) Mean ± SD	High GADS Men (n = 15,578) Mean ± SD	*p*-value	Low GADS Women (n = 41,463) Mean ± SD	High GADS Women (n = 6086) Mean ± SD	*p*-value
Systolic blood pressure (mmHg)	114.6 ± 7.9	131.6 ± 15.0	<0.001	108.7 ± 9.6	122.1 ± 16.8	<0.001
Diastolic blood pressure (mmHg)	69.8 ± 7.9	79.6 ± 10.5	<0.001	66.1 ± 7.8	74.5 ± 11.2	<0.001
Total cholesterol (mg/dL)	185.0 ± 34.4	203.8 ± 40.2	<0.001	182.6 ± 31.9	209.5 ± 36.8	<0.001
HDL-c (mg/dL)	53.2 ± 5.8	49.4 ± 7.3	<0.001	57.1 ± 5.8	49.1 ± 7.4	<0.001
LDL-c (mg/dL)	114.6 ± 34.3	124.5 ± 39.5	<0.001	110.4 ± 31.7	139.4 ± 37.5	<0.001
Triglycerides (mg/dL)	86.1 ± 28.7	151.7 ± 57.9	<0.001	75.4 ± 26.6	105.6 ± 41.9	<0.001
Fasting glucose (mg/dL)	83.3 ± 9.4	91.6 ± 14.0	<0.001	81.4 ± 9.3	87.8 ± 13.3	<0.001
Body mass index (kg/m²)	25.2 ± 3.4	28.1 ± 4.6	<0.001	23.8 ± 4.1	27.0 ± 5.2	<0.001
Waist circumference (cm)	82.4 ± 7.1	91.9 ± 9.8	<0.001	69.1 ± 6.3	79.8 ± 8.2	<0.001

GADS, Goldberg Anxiety and Depression Scale; SD, standard deviation; HDL-c, high-density lipoprotein cholesterol; LDL-c, low-density lipoprotein cholesterol. Continuous variables are expressed as mean ± standard deviation. Comparisons between low and high anxiety groups were performed using Student’s *t*-test. Statistical significance was established at *p* < 0.05. Low GADS: Goldberg Anxiety and Depression Scale score <4 points. High GADS: Goldberg Anxiety and Depression Scale score ≥4 points.

### Prevalence of Cardiometabolic Alterations According to Anxiety Status

The prevalence of cardiometabolic alterations increased substantially among participants with elevated GADS scores.

In men, the prevalence of hypertension increased from 6.9% in the low-anxiety group to 36.9% among workers with elevated anxiety scores. Hypertriglyceridemia increased from 7.9% to 41.3%, whereas impaired fasting glucose rose from 4.9% to 28.0%.

Similarly, women with elevated anxiety exhibited significantly higher prevalences of hypertension, dyslipidemia, hypertriglyceridemia, and impaired fasting glucose compared with those presenting lower anxiety scores.

Particularly notable was the increase in LDL-c abnormalities among women, with prevalence rising from 27.0% in the low-anxiety group to 60.3% in workers with elevated anxiety.

These findings indicate a strong and clinically meaningful association between anxiety symptoms and cardiometabolic dysfunction in occupational populations.

Prevalence analyses are shown in Table 3.

**Table 3.** **Prevalence of cardiometabolic alterations according to Goldberg Anxiety and Depression Scale status and sex**.

Cardiometabolic Alterations	Low GADS Men (n = 56,209) %	High GADS Men (n = 15,578) %	*p*-value	Low GADS Women (n = 41,463)%	High GADS Women (n = 6086) %	*p*-value
Hypertension	6.9	36.9	<0.001	4.5	20.3	<0.001
Total cholesterol ≥200 mg/dL	33.2	53.1	<0.001	27.9	61.2	<0.001
LDL-c ≥130 mg/dL	32.8	56.8	<0.001	27.0	60.3	<0.001
Hypertriglyceridemia (≥150 mg/dL)	7.9	41.3	<0.001	4.9	18.8	<0.001
Low HDL-c	18.1	42.7	<0.001	14.9	39.5	<0.001
Impaired fasting glucose (≥100 mg/dL)	4.9	28.0	<0.001	5.7	18.4	<0.001
General obesity (BMI ≥30 kg/m^2^)	11.8	29.7	<0.001	10.2	26.4	<0.001
Abdominal obesity	16.5	48.6	<0.001	19.1	51.7	<0.001
Metabolic syndrome	8.4	34.8	<0.001	6.9	27.5	<0.001

GADS, Goldberg Anxiety and Depression Scale; HDL-c, high-density lipoprotein cholesterol; LDL-c, low-density lipoprotein cholesterol; BMI, body mass index. Categorical variables are expressed as percentages. Comparisons between low and high anxiety groups were performed using chi-square tests. Metabolic syndrome was defined according to the harmonized international criteria. Statistical significance was established at *p* < 0.05. Low GADS: Goldberg Anxiety and Depression Scale score <4 points. High GADS: Goldberg Anxiety and Depression Scale score ≥4 points.

### Lifestyle Behaviors and Anxiety Profiles

Important differences in lifestyle habits were identified according to anxiety status.

Workers with elevated GADS scores reported: lower levels of regular physical activity, poorer adherence to Mediterranean dietary patterns, and higher smoking prevalence.

Sedentary behavior was particularly frequent among anxious workers and showed strong associations with adverse metabolic parameters. Individuals combining elevated anxiety and low physical activity levels exhibited the highest prevalence of obesity, hypertension, hypertriglyceridemia, and impaired fasting glucose.

Likewise, poor adherence to the Mediterranean diet amplified the unfavorable metabolic profile associated with anxiety. Participants simultaneously presenting high anxiety and low PREDIMED scores showed a higher prevalence of cardiometabolic alterations.

The combined effects of anxiety and lifestyle factors on cardiometabolic alterations are summarized in Fig. 1.

**Fig. 1.**
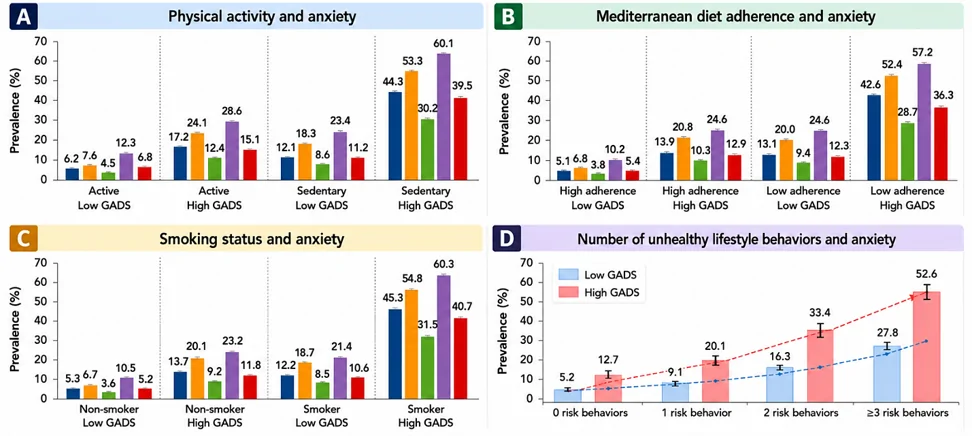
**Combined prevalence of cardiometabolic alterations according to Goldberg Anxiety and Depression Scale status and lifestyle habits**. (A) Physical activity and anxiety. (B) Mediterranean diet adherence and anxiety. (C) Smoking status and anxiety. (D) Number of unhealthy lifestyle behaviors and anxiety. Low GADS: Goldberg Anxiety and Depression Scale score <4 points. High GADS: Goldberg Anxiety and Depression Scale score ≥4 points. Active: moderate or high physical activity according to IPAQ-SF criteria. Sedentary: low physical activity according to IPAQ-SF criteria. High adherence: Prevention with Mediterranean Diet (PREDIMED) score ≥9 points. Low adherence: PREDIMED score <9 points. Risk behaviors included current smoking, physical inactivity, low adherence to the Mediterranean diet, and elevated anxiety symptoms (GADS ≥4).

### Multivariable Logistic Regression Analyses

After adjustment for age, sex, educational level, occupational social class, smoking status, physical activity, and Mediterranean diet adherence, elevated GADS scores remained independently associated with all major cardiometabolic alterations.

High anxiety scores were associated with: hypertension, hypercholesterolemia,elevated LDL-c, hypertriglyceridemia, and impaired fasting glucose.

Among the analyzed outcomes, elevated anxiety showed strong associations with hypertension, hypertriglyceridemia, and impaired fasting glucose. Workers with elevated anxiety had 5.31 times the odds of presenting hypertriglyceridemia compared with individuals with lower anxiety scores.

Similarly, anxiety remained strongly associated with impaired fasting glucose after multivariable adjustment, supporting an independent relationship between anxiety symptoms and altered glucose metabolism.

Physical inactivity was strongly associated with all analyzed cardiometabolic outcomes and showed particularly high odds ratios for hypertriglyceridemia, hypercholesterolemia, and elevated LDL-c. Poor adherence to the Mediterranean diet was independently associated with all adverse lipid parameters and impaired glucose regulation.

Smoking was also significantly associated with worse cardiometabolic profiles, although its effect size was smaller than those observed for anxiety and physical inactivity.

The fully adjusted logistic regression models are presented in Table 4 (Ref. [27, 28, 29, 30]). To improve clarity and facilitate interpretation, the multivariable analyses and graphical presentations were restricted to the major cardiometabolic outcomes showing the greatest clinical relevance and strongest associations with anxiety symptoms, namely hypertension, hypertriglyceridemia, impaired fasting glucose, hypercholesterolemia, and elevated LDL-c.

**Table 4.** **Multivariable logistic regression analysis for cardiometabolic alterations according to anxiety status and lifestyle factors**.

Variables		Hypertension OR (95% CI)	Hypercholesterolemia OR (95% CI)	Elevated LDL-c OR (95% CI)	Hypertriglyceridemia OR (95% CI)	Impaired fasting glucose OR (95% CI)
Sex						
	Women	1 (reference)	1 (reference)	1 (reference)	1 (reference)	1 (reference)
	Men	2.39 (2.29–2.49)	1.28 (1.22–1.34)	1.25 (1.20–1.30)	3.54 (3.38–3.71)	1.79 (1.71–1.88)
Age groups (years)						
	20–29	1 (reference)	1 (reference)	1 (reference)	1 (reference)	1 (reference)
	30–39	1.26 (1.16–1.37)	1.13 (1.09–1.17)	1.18 (1.14–1.23)	1.19 (1.14–1.24)	1.18 (1.14–1.23)
	40–49	1.93 (1.78–2.09)	1.27 (1.22–1.32)	1.50 (1.39–1.61)	1.61 (1.48–1.75)	1.59 (1.46–1.72)
	50–59	2.75 (2.52–2.99)	2.21 (2.05–2.37)	2.60 (2.41–2.80)	2.49 (2.18–2.80)	2.20 (2.01–2.40)
	60–69	3.26 (2.96–3.57)	4.30 (3.96–4.65)	5.43 (5.00–5.87)	3.69 (3.30–4.09)	2.98 (2.68–3.28)
Educational level						
	University	1 (reference)	1 (reference)	1 (reference)	1 (reference)	1 (reference)
	High school	1.14 (1.10–1.19)	1.19 (1.15–1.24)	1.11 (1.08–1.14)	1.23 (1.19–1.28)	1.22 (1.15–1.29)
	Elementary school	1.31 (1.26–1.37)	1.39 (1.30–1.49)	1.46 (1.33–1.59)	1.92 (1.73–2.12)	1.33 (1.26–1.40)
Social class						
	Class I	1 (reference)	1 (reference)	1 (reference)	1 (reference)	1 (reference)
	Class II	1.10 (1.07–1.14)	1.16 (1.13–1.22)	1.14 (1.10–1.19)	1.25 (1.21–1.30)	1.20 (1.16–1.25)
	Class III	1.25 (1.18–1.32)	1.35 (1.27–1.43)	1.52 (1.39–1.65)	1.88 (1.69–2.08)	1.40 (1.33–1.47)
Physical activity						
	Active	1 (reference)	1 (reference)	1 (reference)	1 (reference)	1 (reference)
	Physically inactive	3.89 (3.49–4.30)	4.21 (3.90–4.53)	4.05 (3.85–4.26)	8.25 (6.99–9.51)	3.68 (3.38–3.99)
Mediterranean diet adherence						
	High adherence	1 (reference)	1 (reference)	1 (reference)	1 (reference)	1 (reference)
	Low adherence	1.88 (1.69–2.08)	2.30 (2.11–2.50)	2.33 (2.13–2.54)	3.62 (3.30–3.95)	1.38 (1.27–1.49)
Smoking status						
	Non-smoker	1 (reference)	1 (reference)	1 (reference)	1 (reference)	1 (reference)
	Current smoker	1.13 (1.09–1.17)	1.16 (1.12–1.20)	1.09 (1.06–1.13)	1.21 (1.17–1.25)	1.11 (1.07–1.15)
Goldberg Anxiety and Depression Scale						
	Low anxiety	1 (reference)	1 (reference)	1 (reference)	1 (reference)	1 (reference)
	High anxiety	6.48 (5.89–7.09)	1.98 (1.79–2.18)	2.95 (2.46–3.45)	5.31 (4.60–6.02)	5.23 (4.64–5.83)

OR, odds ratio; CI, confidence interval; LDL-c, low-density lipoprotein cholesterol. Multivariable logistic regression models were adjusted for sex, age, educational level, social class, smoking status, physical activity, Mediterranean diet adherence, and Goldberg Anxiety and Depression Scale status. Statistical significance was established at *p* < 0.05. Active: moderate or high physical activity according to IPAQ-SF criteria. Physically inactive: low physical activity according to IPAQ-SF criteria. High Mediterranean diet adherence: Prevention with Mediterranean Diet (PREDIMED) score ≥9 points. Low Mediterranean diet adherence: PREDIMED score <9 points. Low anxiety: Goldberg Anxiety and Depression Scale score <4 points. High anxiety: Goldberg Anxiety and Depression Scale score ≥4 points [27, 28, 29, 30].

### Adjusted Odds Ratios for Major Cardiometabolic Outcomes

Fig. 2 presents forest plots of the adjusted ORs with their corresponding 95% CIs obtained from the multivariable logistic regression models for hypertension, hypercholesterolemia, elevated LDL-c, hypertriglyceridemia, and impaired fasting glucose. The forest plots facilitate visual comparison of the magnitude and precision of the associations across the five cardiometabolic outcomes. Overall, high GADS scores and physical inactivity exhibited the strongest associations with cardiometabolic alterations, whereas low adherence to the Mediterranean diet and current smoking were also independently associated with increased cardiometabolic risk.

**Fig. 2.**
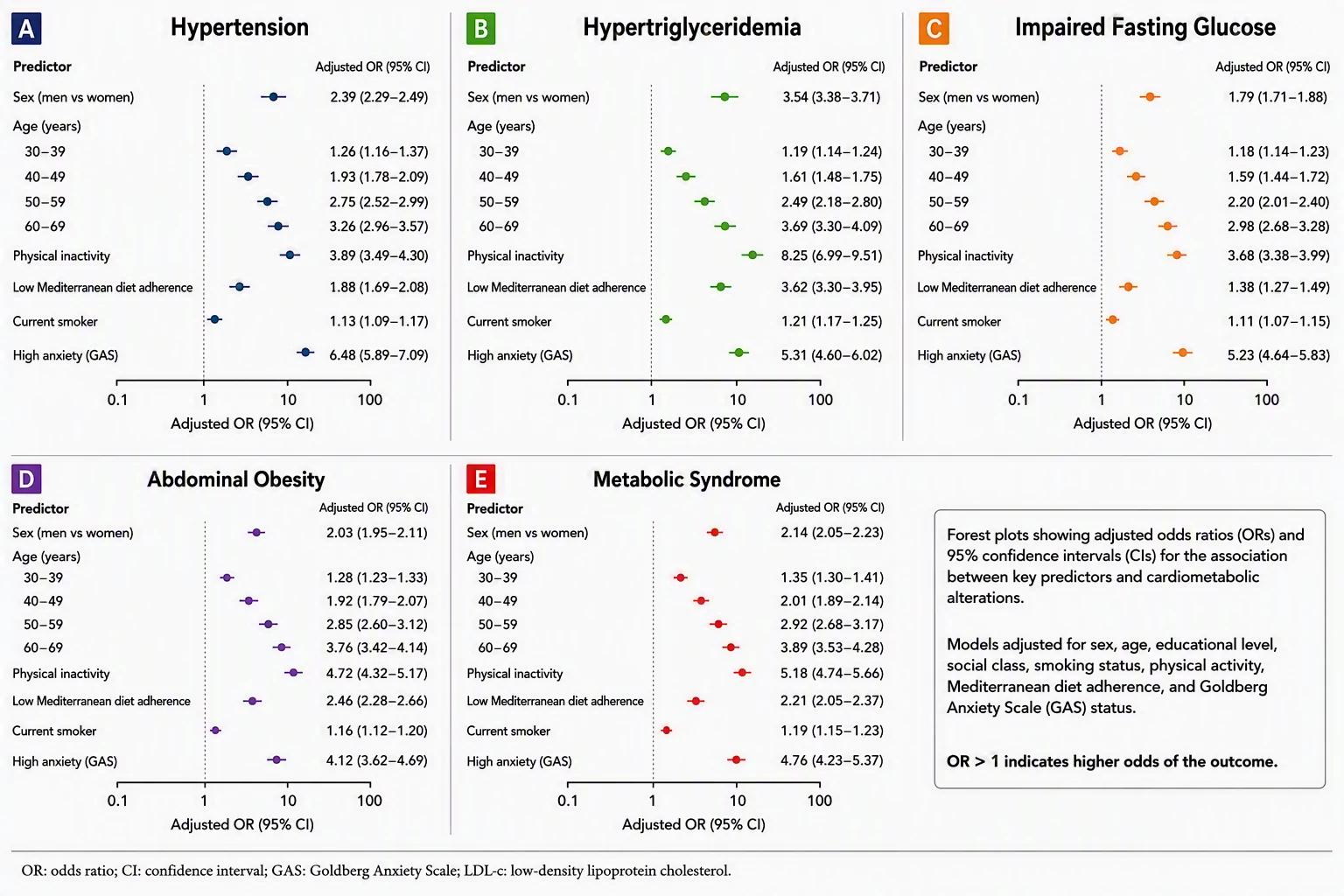
**Forest plots of adjusted odds ratios for major cardiometabolic alterations according to sociodemographic, lifestyle, and psychological factors**. (A) Hypertension. (B) Hypercholesterolemia. (C) Elevated LDL-c. (D) Hypertriglyceridemia. (E) Impaired fasting glucose. Forest plots present adjusted odds ratios (ORs) with 95% confidence intervals (95% CIs) estimated from multivariable logistic regression models. Estimates were adjusted for sex, age, educational level, occupational social class, physical activity, adherence to the Mediterranean diet, smoking status, and Goldberg Anxiety and Depression Scale (GADS) score. The dashed vertical line indicates the null value (OR = 1.0), with estimates to the right representing higher odds relative to the corresponding reference category.

### Sex-Stratified Analyses

Sex-specific analyses revealed important differences in the relationship between anxiety and cardiometabolic risk.

In men, elevated anxiety scores were more strongly associated with hypertension, hypertriglyceridemia, impaired fasting glucose, and metabolic syndrome. Conversely, women showed a relatively stronger association between anxiety and abdominal obesity.

Despite these differences, anxiety remained a consistent independent predictor of adverse cardiometabolic outcomes in both sexes.

These findings support the existence of partially distinct biological and behavioral pathways linking anxiety and metabolic dysfunction in men and women.

Sex-stratified regression analyses are summarized in Table 5.

**Table 5.** **Sex-stratified multivariable logistic regression analyses for cardiometabolic alterations according to Goldberg Anxiety and Depression Scale status and lifestyle factors**.

Variables		Men OR (95% CI)	Women OR (95% CI)	*p* for interaction
Hypertension				
	Goldberg Anxiety and Depression Scale score	6.92 (6.18–7.76)	5.41 (4.73–6.18)	<0.001
	Physical inactivity	4.11 (3.78–4.47)	3.54 (3.18–3.94)	0.003
	Mediterranean diet adherence	1.95 (1.79–2.12)	1.74 (1.59–1.91)	0.012
	Current smoking	1.17 (1.11–1.23)	1.08 (1.02–1.15)	0.041
Hypertriglyceridemia				
	Goldberg Anxiety and Depression Scale score	5.88 (5.17–6.69)	4.46 (3.88–5.12)	<0.001
	Physical inactivity	8.74 (7.61–10.03)	6.92 (5.89–8.13)	<0.001
	Mediterranean diet adherence	3.81 (3.46–4.20)	3.02 (2.71–3.36)	0.005
	Current smoking	1.26 (1.20–1.33)	1.14 (1.07–1.22)	0.027
Impaired fasting glucose				
	Goldberg Anxiety and Depression Scale score	5.63 (4.98–6.35)	4.61 (4.01–5.29)	<0.001
	Physical inactivity	3.84 (3.49–4.22)	3.19 (2.83–3.58)	0.008
	Mediterranean diet adherence	1.43 (1.31–1.56)	1.29 (1.17–1.42)	0.034
	Current smoking	1.12 (1.06–1.19)	1.07 (1.00–1.14)	0.083
Abdominal obesity				
	Goldberg Anxiety and Depression Scale score	4.35 (3.88–4.88)	4.98 (4.39–5.66)	0.006
	Physical inactivity	4.88 (4.39–5.42)	4.52 (4.01–5.10)	0.094
	Mediterranean diet adherence	2.51 (2.29–2.75)	2.34 (2.12–2.59)	0.115
	Current smoking	1.19 (1.13–1.25)	1.11 (1.04–1.19)	0.038
Metabolic syndrome				
	Goldberg Anxiety and Depression Scale score	5.02 (4.43–5.68)	4.31 (3.77–4.93)	0.011
	Physical inactivity	5.46 (4.87–6.12)	4.77 (4.16–5.46)	0.019
	Mediterranean diet adherence	2.36 (2.14–2.61)	2.08 (1.86–2.33)	0.028
	Current smoking	1.22 (1.15–1.30)	1.13 (1.05–1.22)	0.044

OR, odds ratio; CI, confidence interval. Sex-stratified multivariable logistic regression models were adjusted for age, educational level, occupational social class, smoking status, physical activity, Mediterranean diet adherence, and GADS status. Odds ratios are presented separately for men and women. *p* values for interaction evaluate sex-related differences in the association between lifestyle factors, anxiety, and cardiometabolic outcomes. Statistical significance was established at *p* < 0.05.

## Discussion

### Principal Findings

The present study provides a comprehensive evaluation of the relationship between anxiety symptoms, lifestyle behaviors, and cardiometabolic risk in a very large cohort of Spanish workers. The findings demonstrate that elevated GADS scores are consistently associated with a markedly less favorable cardiometabolic profile, including higher prevalence of hypertension, dyslipidemia, hypertriglyceridemia, impaired fasting glucose, abdominal obesity, and metabolic syndrome. Importantly, these associations persisted after adjustment for major sociodemographic and lifestyle-related confounders, suggesting that anxiety may represent an independent contributor to cardiometabolic dysfunction.

The results additionally reveal that anxiety symptoms and unhealthy lifestyle habits frequently co-occur and are jointly associated with a greater cardiometabolic burden. Workers presenting elevated anxiety levels together with physical inactivity, smoking, or poor adherence to the Mediterranean diet exhibited the highest cardiometabolic burden observed in the cohort.

Taken together, these findings suggest that anxiety symptoms are associated with a less favorable cardiometabolic profile and may serve as a marker of increased cardiometabolic vulnerability in occupational populations.

A key strength and novel aspect of this study is the simultaneous evaluation of anxiety symptoms, lifestyle behaviors, and a broad range of cardiometabolic outcomes within a very large occupational cohort. Unlike many previous studies that focused on single metabolic outcomes or specific clinical populations, our analysis provides a comprehensive assessment of the interrelationships between psychological, behavioral, and cardiometabolic factors in routine occupational health settings.

### Comparison With Previous Literature

The association between anxiety and adverse cardiometabolic outcomes observed in the present study is consistent with a substantial body of epidemiological evidence linking psychological distress with cardiovascular disease, obesity, insulin resistance, and metabolic syndrome [39, 40, 41, 42]. Several studies have shown that anxiety symptoms are associated with increased risk of hypertension, coronary heart disease, type 2 diabetes, and cardiovascular mortality, even after adjustment for traditional cardiovascular risk factors [5, 42, 43, 44].

Our findings are also aligned with previous occupational studies suggesting that psychosocial stress and anxiety contribute significantly to metabolic impairment among working populations [45, 46]. In particular, the markedly higher prevalence of hypertriglyceridemia and impaired fasting glucose among workers with elevated anxiety scores aligns with previous findings suggesting that anxiety and psychological stress may contribute to cardiometabolic dysfunction through alterations in lipid metabolism, glucose regulation, and the development of metabolic syndrome [8, 47, 48].

The strong association between anxiety and abdominal obesity identified in our analyses is especially relevant. Central adiposity has been increasingly recognized as one of the major biological links between psychological stress and cardiometabolic disease [49]. Visceral fat accumulation is closely related to chronic low-grade inflammation, insulin resistance, endothelial dysfunction, and autonomic imbalance, all of which may contribute to the development of cardiovascular disease [50, 51].

Our results also support the growing evidence highlighting the importance of lifestyle behaviors in the association between anxiety symptoms and cardiometabolic risk. Physical inactivity, smoking, and unhealthy dietary patterns were substantially more frequent among workers with elevated anxiety scores, which is consistent with previous literature indicating that anxious individuals often adopt maladaptive coping behaviors that further worsen metabolic health [52, 53].

Importantly, our findings are also coherent with previous research conducted in Spanish occupational populations using the GADS. Vicente-Herrero *et al*. [54] recently demonstrated a significant association between GADS scores and adverse cardiometabolic indicators in more than 119,000 Spanish workers, highlighting the potential relevance of anxiety screening within occupational health programs. The present study extends these observations by incorporating detailed analyses of lifestyle behaviors within a large occupational cohort.

### Potential Mechanisms

Several biological and behavioral mechanisms may explain the relationship between anxiety and cardiometabolic dysfunction observed in this study.

From a neuroendocrine perspective, chronic anxiety is associated with sustained activation of the hypothalamic–pituitary–adrenal axis and the sympathetic nervous system, resulting in increased cortisol secretion, catecholamine release, and autonomic dysregulation [55, 56]. Prolonged exposure to these stress-related hormonal pathways may promote visceral adiposity, insulin resistance, endothelial dysfunction, and systemic inflammation.

Anxiety has also been linked to increased inflammatory activity, including elevated concentrations of interleukin-6, tumor necrosis factor-alpha, and C-reactive protein, which are themselves strongly associated with atherosclerosis and metabolic syndrome [57, 58]. These inflammatory pathways may partially explain the higher prevalence of hypertension, dyslipidemia, and impaired fasting glucose among anxious workers.

Behavioral mechanisms are likely to play an additional central role. Individuals with anxiety symptoms frequently exhibit poorer sleep quality, lower levels of physical activity, emotional eating behaviors, increased tobacco consumption, and reduced adherence to healthy dietary patterns [59]. In the present study, physically inactive workers with elevated anxiety showed the most adverse cardiometabolic profiles, indicating that the coexistence of these factors is associated with a less favorable cardiometabolic profile.

The Mediterranean diet appears particularly relevant within this context. Participants with both elevated anxiety and low adherence to Mediterranean dietary patterns exhibited substantially worse lipid and glycemic profiles. Previous studies have demonstrated that Mediterranean diet adherence is associated not only with improved cardiovascular health but also with better mental health outcomes and lower levels of psychological distress [60, 61].

Sex-related biological differences may additionally contribute to the observed findings. Men showed stronger associations between anxiety and hypertension or hypertriglyceridemia, whereas women exhibited relatively stronger relationships between anxiety and dyslipidemia or abdominal obesity. Biological and behavioral differences between men and women, including hormonal regulation and fat distribution patterns, may contribute to the sex-specific associations observed in our study [62, 63].

### Clinical and Public Health Implications

The findings of this study have several important clinical and public health implications.

First, they support the incorporation of mental health assessment into cardiovascular and metabolic prevention programs, particularly within occupational health settings. Anxiety screening using brief validated tools such as the GADS may help identify individuals at increased cardiometabolic risk who could otherwise remain undetected.

Second, the results highlight the need for integrated interventions simultaneously targeting psychological well-being and lifestyle modification. Traditional cardiometabolic prevention strategies often focus exclusively on diet, exercise, or smoking cessation without adequately addressing underlying psychological distress. Our findings suggest that anxiety may substantially compromise the effectiveness of these interventions if left untreated.

Third, workplace health promotion programs may represent an especially valuable opportunity for integrated prevention. Occupational settings provide direct access to large adult populations during economically productive years, facilitating the implementation of multidimensional interventions combining mental health support, physical activity promotion, smoking cessation, and nutritional counseling.

Finally, these findings reinforce the broader concept that mental health and cardiometabolic health are deeply interconnected and should not be managed as independent clinical domains. Public health strategies aimed at reducing cardiovascular burden may therefore benefit from incorporating psychological and psychosocial dimensions into prevention policies.

The large sample size and occupational setting enhance the practical applicability of these findings for workplace prevention programs and population-based cardiometabolic risk assessment strategies.

### Strengths and Limitations

This study has several important strengths. It includes a very large occupational cohort with more than 119,000 participants, allowing robust statistical analyses and detailed sex-stratified evaluations. Standardized clinical, anthropometric, biochemical, and lifestyle assessments were systematically performed under occupational health surveillance conditions, improving data consistency and reliability.

The simultaneous evaluation of anxiety, lifestyle habits, and multiple cardiometabolic outcomes additionally provides a multidimensional perspective rarely available in occupational epidemiology studies.

Nevertheless, several limitations should also be acknowledged. The cross-sectional design precludes establishing causal relationships, and reverse causality cannot be excluded. Anxiety symptoms were assessed using a validated screening questionnaire rather than formal psychiatric diagnostic interviews. Although the GADS has demonstrated good sensitivity and specificity in epidemiological settings, some degree of misclassification remains possible.

Residual confounding may additionally persist despite multivariable adjustment, particularly regarding unmeasured psychosocial variables, sleep disorders, psychotropic medication use, other pharmacological treatments, or socioeconomic stressors. Information regarding the use of anxiolytics, antidepressants, or other psychotropic medications was not available and therefore could not be considered in the analyses. Given the potential metabolic effects of these medications, residual confounding cannot be completely excluded. In addition, former smokers were classified within the non-smoker category because information regarding smoking cessation duration was not consistently available. This approach may have introduced misclassification bias and could have attenuated the observed associations between smoking status and cardiometabolic outcomes. Furthermore, the study population consisted exclusively of actively employed workers, which may limit extrapolation to unemployed or clinically vulnerable populations.

Although multiple statistical comparisons were performed, the principal findings were highly consistent across descriptive analyses, multivariable regression models, and sex-stratified analyses, with most associations showing strong statistical significance (*p* < 0.001). Nevertheless, as in any study involving multiple statistical comparisons, the possibility of type I error cannot be completely excluded.

Future longitudinal studies should evaluate potential mediation pathways through which lifestyle behaviors may influence the association between anxiety symptoms and cardiometabolic risk.

Despite these limitations, the consistency of the findings across multiple analyses and outcomes strongly supports the existence of a clinically meaningful relationship between anxiety and cardiometabolic dysfunction.

## Conclusions

In conclusion, elevated GADS scores are strongly associated with adverse cardiometabolic profiles in Spanish workers, particularly when combined with unhealthy lifestyle behaviors such as physical inactivity, smoking, and poor adherence to the Mediterranean diet. Anxiety showed independent associations with hypertension, hypertriglyceridemia, impaired fasting glucose, abdominal obesity, and metabolic syndrome.

These findings reinforce the need for integrated prevention strategies simultaneously addressing mental health, lifestyle behaviors, and cardiometabolic risk within occupational and public health settings.

## Availability of Data and Materials

The data supporting the findings of this study are stored in a secure database maintained by ADEMA University School and are available from the corresponding author upon reasonable request and subject to institutional data protection regulations. The Data Protection Officer responsible for data oversight is Ángel Arturo López González.
